# Genetic Impairment of Cellulose Biosynthesis Increases Cell Wall Fragility and Improves Lipid Extractability from Oleaginous Alga *Nannochloropsis salina*

**DOI:** 10.3390/microorganisms8081195

**Published:** 2020-08-06

**Authors:** Seok Won Jeong, Kwon HwangBo, Jong Min Lim, Seung Won Nam, Bong Soo Lee, Byeong-ryool Jeong, Yong Keun Chang, Won-Joong Jeong, Youn-Il Park

**Affiliations:** 1 Department of Biological Sciences, Chungnam National University, Daejeon 34134, Korea; neditbe@cnu.ac.kr; 2 Korea Research Institute of Bioscience and Biotechnology, Daejeon 34141, Korea; rnjsl@kribb.re.kr (K.H.); jmlim114@kribb.re.kr (J.M.L.); 3 Bioresouces Culture Collection Division, Nakdonggang National Institute of Biological Resources, Sangju 37242, Korea; seungwon10@nnibr.re.kr; 4 Department of Microbial and Nano Materials, College of Science and Technology, Mokwon University, Daejeon 35349, Korea; bongsoolee@mokwon.ac.kr; 5 Single-Cell Center, CAS Key Laboratory of Biofuels and Shandong Key Laboratory of Energy Genetics, Qingdao Institute of BioEnergy and Bioprocess Technology (QIBEBT), Qingdao 266101, China; bjeong@unist.ac.kr; 6 School of Energy and Chemical Engineering, Ulsan National Institute of Science and Technology (UNIST), Ulsan 44919, Korea; 7 Department of Chemical and Biomolecular Engineering, Korea Advanced Institute of Science and Technology, Daejeon 34141, Korea; changyk@kaist.ac.kr

**Keywords:** cell wall, cellulose synthase CesA, *cesA* mutant, CRISPR/Cas9, photosynthate partitioning, *Nannochloropsis*

## Abstract

In microalgae, photosynthesis provides energy and sugar phosphates for the biosynthesis of storage and structural carbohydrates, lipids, and nitrogenous proteins. The oleaginous alga *Nannochloropsis salina* does not preferentially partition photoassimilates among cellulose, chrysolaminarin, and lipids in response to nitrogenous nutrient deprivation. In the present study, we investigated whether genetic impairment of the cellulose synthase gene (*CesA*) expression would lead to protein accumulation without the accumulation of storage C polymers in *N. salina*. Three *cesA* mutants were generated by the CRISPR/Cas9 approach. Cell wall thickness and cellulose content were reduced in the *cesA1* mutant, but not in *cesA2* or *cesA4* cells. *CesA1* mutation resulted in a reduction of chrysolaminarin and neutral lipid contents, by 66.3% and 37.1%, respectively, but increased the soluble protein content by 1.8-fold. Further, *N. salina* cells with a thinned cell wall were susceptible to mechanical stress, resulting in a 1.7-fold enhancement of lipid extractability. Taken together, the previous and current studies strongly suggest the presence of a controlling mechanism that regulates photoassimilate partitioning toward C and N metabolic pathways as well as the cellulose metabolism as a potential target for cost-effective microalgal cell disruption and as a useful protein production platform.

## 1. Introduction

In microalgae, similar to other photoautotrophs, the energy and building blocks used in the C, N, and S metabolism, such as carbohydrates, lipids, nucleic acids, and proteins, are derived from photosynthetic electron transport. Fixed C, assimilated via the Calvin cycle, is subsequently allocated to cell growth, maintenance, and division. In response to the changing environment, the energy flux provided by photosynthesis is promptly adjusted by the C flux for macromolecule synthesis, which is controlled by efficient regulatory mechanisms. For instance, under nutrient-rich conditions, fixed C is mostly routed into proteins. On the other hand, N limitation favors C allocation that (frequently) leads to increased carbohydrate fraction and a reduced protein fraction. Consequently, the photosynthetic apparatus is forced to deal with excess photons, since carbohydrate synthesis requires fewer electrons per C than protein synthesis. Hence, understanding this regulatory mechanism is important in terms of the biotechnological and ecological aspects of algae [1].

*Nannochloropsis* sp., unicellular and nonmotile microalgae, are oleaginous, with high lipid productivity [2,3,4,5]. *Nannochloropsis* produces neutral triacylglycerol lipids (up to 60% of the cell dry weight (CDW)) and polyunsaturated fatty acids, such as eicosapentaenoic acid, which can be used as feedstock for biodiesel conversion and as a functional food, respectively [6]. Among nine *Nannochloropsis* strains analyzed to date, the high biomass productivity and salinity resistance of *N. salina* favor commercial applications that involve cultivation with two-stage N deprivation [7,8]. Further, the low lipid content of *N. salina* is a potential target for biotechnological improvement of lipid productivity [9]. In *N. salina*, similar to other algae including *Chlamydomonas reinhardtii* [10], N limitation favors C allocation to C metabolism at the expense of protein synthesis. In addition to lipid accumulation, production of chrysolaminarin and cellulose is simultaneously enhanced because of transcriptional activation of several genes encoding biosynthetic enzymes for these carbohydrates [9]. This strongly indicates that N limitation regulates photoassimilate partitioning between the storage (lipid and chrysolaminarin) and structural (cellulose) C macromolecules on the transcriptional level, according to an equal allocation principle, a process that is not well understood. Further, N-induced cell wall thickening results in cells that are more prone to mechanical stress than control cells [9].

Cell wall thickness in *Nannochloropsis* species depends on distinct genetic traits or concentrations of nutrients such as salt, N, P, and S [6,9,11,12]. Like in other algae, the cell wall of *N. salina* contains carbohydrates, proteins, and lipids. Cellulose is the major cell wall polysaccharide in *Nannochloropsis* species (for instance, it accounts for 75% of all polysaccharides in *N. gaditana* [13] and 80% in *N. oculata* [14]) and forms the inner layer of the cell wall bilayer. The outer wall contains layers of long-chain aliphatic hydrocarbons such as C_28_–C_34_ algaenan, which is similar to cutan [13]. Despite the abundance and plasticity of cellulose component in response to varying nutrient concentrations, genetic manipulation targeting cell wall thickness by controlling cellulose biosynthesis has not yet been reported.

In the present study, we tested the fine regulatory mechanism that seesaws the overall flow of photoassimilates between C polymers and proteins in a reciprocal manner in *N. salina*. We expected that blocking of at least one branched pathway leading to the production of these C polymers would foster protein accumulation. Here, by using targeted mutagenesis, we impaired the cellulose biosynthesis pathway, which has bearing on the biofuel production process, including algal growth, harvesting, dewatering, and extraction [12,13,15]. Cellulose biosynthesis in *N. salina* is initiated by the formation of UDP-glucose (UDP-Glc) from Glc-6 phosphate (Glc-6-P) by UDP-Glc pyrophosphorylase (UPP), followed by cellulose biosynthesis by CesA enzymes that utilize UDP-Glc and *β*-1,4-glucan [4,13]. Further, in *N. salina*, *CesA* genes are transcriptionally activated by N-deprivation [9]. In the current study, we used RNA interference (RNAi) or CRISPR/Cas9 approaches to partially or fully inhibit the expression of three (CesA1, 2, and 4-encoding) genes, respectively. The generated *cesA1* knockout (KO) mutants were characterized by a reduced cell wall thickness, and reduced soluble carbohydrate chrysolaminarin and storage lipid contents, with enhanced protein content and reactive oxygen species (ROS) levels. Furthermore, *cesA1* KO of *N. salina* with a thinned cell wall was more susceptible to mechanical disruption than the wild-type (WT) cells, enabling improved lipid extraction efficiency compared with that of the control cells. Therefore, reciprocal regulation of the C and N anabolic pathways offers new directions for a rational design of *N. salina* into biofuel feedstock, as well as a useful protein production reactor.

## 2. Materials and Methods

### 2.1. Culture Conditions

*N. salina* CCMP1776 cells were maintained on agar plates or cultivated in a modified f/2 medium containing 30 g L^−1^ sea salts and bubbled with 5% (*w/w*) CO_2_ under fluorescent illumination (50–80 μmol m^−2^/s^−1^) with a photoperiod of 16 h light/8 h dark at 28 °C [9]. N-deficiency was induced in cells in the exponential growth phase by washing twice with a growth medium lacking NaNO_3_. To block *de novo* translation, cells were treated with 250 μg mL^−1^ cycloheximide for 24 or 48 h. Algal growth was monitored spectrophotometrically by determining OD_750_ (Shimadzu UV 1800, Kyoto, Japan), and cells were counted using a Bürker counting chamber (Marienfeld-Superior, Lauda-Königshofen, Germany), under a light microscope.

### 2.2. Generation of cesA KO and Knockdown (KD) Mutants

The CRISPR/Cas9 system was used to generate *cesA* KO mutants. A *cas9* gene cut out using NcoI and BamHI enzymes from pCAMBIA-Cas9 vector [16] was cloned into pNs301 vector [17] to generate pNsCas9 vector (Supplementary Figure S1). DNA fragments encoding CesA proteins and including restriction enzyme sites in a region corresponding to the N-terminus of target protein, and the PAM sequence, U6 promoter, and U6 terminator of *N. salina*, were PCR-amplified using specific primers (Supplementary Table S1) and cloned into pBSKSII to generate sgRNA expression vectors (Supplementary Figure S1). As selection marker genes, bleomycin resistance *shble* and codon-optimized *nptII* were used for *cas9* and sgRNA expression, respectively. For *cesA* KD mutants, fragments corresponding to the C-terminal regions of CesA1–4 proteins were amplified from the *N. salina* cDNA by PCR using gene-specific primers (Supplementary Table S1). They were then digested with BamHI and EcoRV for the sense construct, and HindIII and XhoI for the antisense construct, and ligated in sense and antisense orientations, accordingly, to generate pNsCesAs-RNAi vectors (Supplementary Figure S1). Transformants were selected based on the presence of *shble*. Cells of *N. salina* CCMP1776 were transformed with the CAS9/sgRNA vectors and RNAi vectors by electroporation [18]. Cells resistant to 2.5 mg L^−1^ zeocin (Invitrogen, Waltham, MA, USA) and/or 100 mg L^−1^ geneticin (Thermo Fisher Scientific, Waltham, MA, USA) were selected and analyzed. For Southern and Northern analysis, standard protocols [18] were used. Further, *CesA* expression in KD cells was evaluated by Northern analysis using PCR-amplified 0.25 kb fragments corresponding to the *CesA1–4* as probes (Supplementary Table S1).

### 2.3. Growth Rate Determination, Pigment Content Analysis, and Chlorophyll Fluorescence Measurement

Specific growth rate (μ) was calculated according to the equation μ = ln (N2/N1)/(t2 − t1), where N2 and N1 are the total numbers of cells ml^−1^ at the time points t2 and t1, respectively, and where t2 > t1 [9]. Cells (1.2 × 10^8^) were harvested by centrifugation at 3500× *g* for 10 min, washed three times, and then resuspended in deionized water. After lyophilization, CDW was measured [9]. Total chlorophyll *a* and carotenoids were extracted from cells using *N*,*N*′-dimethylformamide [19], and their concentrations were determined spectrophotometrically [20]. Changes in *in vivo* Chl fluorescence in dark-adapted algal cells were monitored by Xe-pulse amplitude-modulated fluorometry (Walz, Effeltrich, Germany) [9]. The *F*_v_/*F*_m_ value, which is an indicator of the maximum PSII efficiency, was calculated as (*F*_m_ − *F*_0_)/*F*_m_, where F_v_ is the dark-adapted variable fluorescence, *F*_m_ is the maximum fluorescence, and F_0_ is the dark-adapted fluorescence. Nonphotochemical quenching parameter (NPQ) was calculated as 1 − *F*/*F*_m’_, where *F* is a steady-state fluorescence and *F*_m’_ is a maximal fluorescence under actinic light illumination. Photosynthetic O_2_ exchange rates in dark-adapted cells were measured using an O_2_ electrode (Hansatech LTD, Norfolk, UK) under varying light intensities obtained using neutral-density filters.

### 2.4. Light, Fluorescence, and Electron Microscopy

Cellulose and neutral lipids were observed by LSM 800 confocal microscopy (Carl Zeiss, Oberkochen, Germany) after cell staining with 0.1% (*w/v*) calcofluor white (CW) in phosphate buffer at pH 6.0 [21] and 0.0025% (*w/v*) Nile red [22], respectively. For ROS detection, cells were diluted to OD_600_ = 0.1 using fresh growth medium containing 5 μM 2′,7′-dichlorodihydrofluorescein diacetate (DCFH-DA) [23]. Confocal images were acquired and processed using ZEN 2.1 Lite software. CW, Nile red, and DCFH-DA fluorescence were also quantified using a spectrofluorometer (LS55; PerkinElmer, Waltham, MA, USA) [9]. Cells were prepared for electron microscopy as previously described. Briefly, cells were fixed in a 1:1 mixture of 2% OsO_4_ and 3% ferrocyanide (*w/v*), dehydrated in a graded ethanol series, and immersed and polymerized in 100% resin at 70 °C. Polymerized sections were stained with 3% (*w/v*) uranyl acetate and Reynold’s lead citrate [24], and then examined and photographed under a JEM-1010 transmission electron microscope operated at 80 kV (JEOL, Tokyo, Japan). Images were recorded on Kodak EM film 4489 (Eastman Kodak Co., New York, NY, USA) and scanned to tagged image file (TIF) format using an Epson Perfection V700 photo scanner (Epson Korea Co., Ltd., Seoul, Korea). Cell wall thickness was analyzed using ImageJ2 (https://imagej.net/ImageJ); cell walls from 250 individual cells were measured in five separate places [9].

### 2.5. Cell Breakage, Cell Wall Extraction, and Cellulose and Chrysolaminarin Quantification

Lyophilized cells (50 mg CDW) were subjected to bead beating with a bead beater (bead diameter: 0.1 mm; BioSpec Products, Bartlesville, OK, USA), and proteins and lipids were removed by hot phenol (70 °C) and chloroform extraction [9]. Cell wall extracts were then washed three times in distilled H_2_O. Soluble and insoluble chrysolaminarin fractions were hydrolyzed into Glc using *β*-1,3-glucanase (No. 61340; Sigma, St. Louis, MO, USA) at 37 °C for 2 h. Insoluble cell wall precipitates were treated with 1% (*w/w*) cellulase (Sigma Cat. No. 61340) isolated from *Aspergillus niger* for 24 h. After centrifugation at 3500× *g* for 10 min, 50 μL supernatant was mixed with 950 μL of 0.2% (*w/v*) anthrone solution in 95% sulfuric acid and boiled for 10 min before measuring OD_620_. Glc was used as a standard to quantify the total cellulose content.

### 2.6. RNA Extraction, cDNA Synthesis, and Quantitative Real-Time PCR (RT-qPCR)

Total RNA was extracted from 200 mg wet biomass using NucleoZol (Macherey-Nagel, Dueren, Germany). DNA was removed with DNase I (Macherey-Nagel)), and cDNA was synthesized using the iScript cDNA synthesis kit (Bio-Rad, Hercules, CA, USA). RT-qPCR was performed using the CFX96 Real-Time System (Bio-Rad) [9]. Primers for PCR reactions were designed to analyze the expression levels of selected key genes (Supplementary Table S2). The housekeeping gene ubiquitin was used as an internal standard. Total RNA and cDNA quantities were determined using the NanoDrop spectrophotometer (Thermo Fisher Scientific, Waltham, MA, USA). Gene expression was calculated using the 2^−∆∆Ct^ method using the CFX Manager program (Bio-Rad).

### 2.7. Cell Breakage, Lipid Extraction Efficiency, Analysis of Fatty Acid Methyl Esters (FAME), and Viability Assays

Cells were broken by using a sonicator (Sonic and Materials, Newtown, CT, USA), two times, at a resonance of 40 W, and with a 5 s operation and 5 s interval cycle. Then, the cells were plated on a fresh f/2 growth medium supplemented with 1.5% (*w/v*) agar and incubated at 22 °C. Colonies were counted after 25 d [9]. The efficiency of cell disruption was expressed as cell viability (determined based on cell numbers) before and after the disruption. Total lipids were extracted using a modified method [25], and the amount of lipids extracted by 20 s sonication was determined after evaporation of chloroform. For FAME analysis, lipids were transesterified, with pentadecane as an internal standard, as described previously [9], and analyzed using gas chromatograph (YL-6100GC; YoungLin Science, Anyang, Korea) equipped with an FID and INNOWAX capillary column (30 m × 0.32 mm × 0.5 μm; Agilent Technologies, Santa Clara, CA, USA). Supelco37 Component Fatty Acid Methyl Ester Mix (Sigma) was used for the identification and quantification of each FAME component, and methyl tridecanoate (C1_3_Me) was used as the recovery standard.

### 2.8. Metabolic Profiling

Three days after inoculation, culture flasks were placed on ice, and cell cultures were transferred to 50 mL centrifuge tubes. After centrifugation at 3500× *g* at 4 °C for 20 min, the pellets were washed twice by vortex-mixing for 30 s, with 10 mL Milli-Q water. The pellets were then mixed with 1.6 mL methanol and ultrasonicated for 30 s to completely suspend them. Then, 1.1 mL of an internal standard solution was added to each tube and mixed well. The tubes were incubated at room temperature for 30 s. After centrifugation at 3000 rpm for 20 min at 4 °C, the supernatants were transferred to ultracentrifugal filter units (Ultrafree-MC-PLHCC-HMT; Human Metabolome Technologies Inc., Yamagata, Japan) and centrifuged at 10,000 rpm at 4 °C for 5 h until no liquid remained in the filter cup. The extracts were evaporated under vacuum at 1500 rpm for 3 h at room temperature. After complete drying, the extracted samples were stored at −80 °C until capillary electrophoresis/time-of-flight mass spectrometry (CE-TOF-MS) analysis (Agilent Technologies). Rapid quenching of metabolic activity by sample freezing was not performed so as to separate intracellular metabolites from those in the growth medium by centrifugation after additional pellet washing.

Metabolites in the extracted samples were analyzed using a CE-TOF/MS system at Human Metabolome Technologies. Cationic metabolites were analyzed using an Agilent CE-TOF-MS system with a fused silica capillary (50 μm × 80 cm). The following conditions were used: run buffer: cation buffer solution (p/n: H3301-1001); rinse buffer: cation buffer solution (p/n: H3301-1001); sample injection: pressure injection 50 mbar, 10 s; CE voltage: positive, 27 kV; MS ionization: ESI positive; MS capillary voltage: 4000 V; MS scan range: *m*/*z* 50–1000; and sheath liquid: HMT sheath liquid (p/n: H3301-1020). Anionic metabolites were analyzed using the same Agilent CE-TOF-MS system with a fused silica capillary (50 μm × 80 cm). The following conditions were used: run buffer: anion buffer solution (p/n: I3302-1023); rinse buffer: anion buffer solution (p/n: I3302-1023); sample injection: pressure injection 50 mbar, 25 s; CE voltage: positive, 30 kV; MS ionization: ESI negative; MS capillary voltage: 3500 V; MS scan range: *m*/*z* 50–1000; and sheath liquid: HMT sheath liquid (p/n: H3301-1020).

Peaks detected during the CE-TOF/MS analysis were extracted using automatic integration software (MasterHands ver. 2.17.1.11 developed at Keio University) to obtain peak information, such as *m/z*, migration time, and peak area. The peak area was then converted to relative peak area by dividing by an internal standard peak area proportional to the sample amount.

### 2.9. Statistical Analysis

All data are expressed as the mean ± standard error (SE) from three or four independent replicates of each experiment. Data were analyzed by two-tailed *t*-test after normality assessment; *p*-values < 0.05 were considered statistically significant.

## 3. Results

### 3.1. Generation of cesA KO and KD Mutants

Among the four *CesA* genes in *N. salina* genome, full-length cDNAs for *CesA1*, *2*, and *4* are identified but only partial cDNA for *CesA3* is present (http://nandesyn.single-cell.cn). To produce the *cesA*s mutant lines, we first constructed KO and KD vectors using Crispr/Cas and RNAi systems, respectively (Supplementary Figure S1). After transformation of cells with vectors containing *Cas9* gene and sgRNA sequences for *cesA1-4*, we successfully generated heritable indel mutations for *cesA1* and *cesA4* (Supplementary Figure S2a,b). However, we were unable to generate *cesA2* or *cesA3* KO lines. Along with the indel mutation, we also attempted to construct *cesA* KD lines. After transformation with RNAi vectors (Supplementary Figure S1), we obtained KD lines for *cesA1*, *cesA2*, and *cesA4* that exhibited lowered expression of target gene, as determined by Northern analysis (Supplementary Figure S2c,d). We were unable to generate KO or KD lines for *cesA3* using the Crispr/Cas9 or RNAi systems, accordingly. In fact, the levels of *CesA3* transcript in WT cells were very low (Supplementary Figure S2d). In the present study, we used two *cesA1* KO lines, *cesA1-38* with 3 bp deletion (mutation resulting in the removal of one amino acid) and *cesA1-111* with 5 bp insertion (nonsense mutation); one *cesA2* KD line (*cesA2-49*); and one *cesA4* KO line, *cesA4-12* with 26 bp deletion (nonsense mutation).

### 3.2. cesA1 Mutant Exhibits Thinned Cell Wall

**Figure 1:**
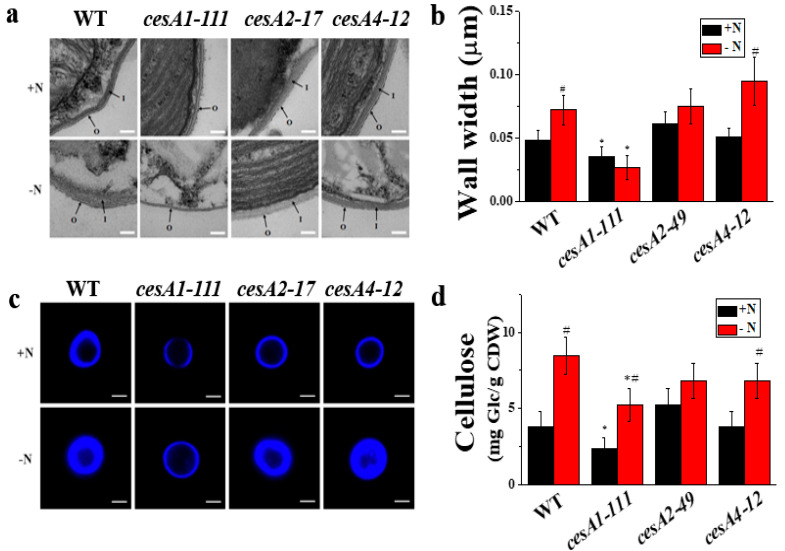
Transmission electron microscopy images of the cell wall (**a**), cell wall width (**b**), calcofluor white (CW) fluorescence staining (**c**), and cellulose content (**d**) of *N. salina* wild-type (WT) and *cesA* mutants grown under +N and –N conditions for 3 d. In (a), I and O represent the inner and outer cell wall layers, respectively. In (**b**), the cell wall of 200 +N or –N cells was measured in five different places. Each data point represents the mean ± SE of three biological replicates performed in triplicate. The symbols represent statistically significant differences (*p* ≤ 0.05) between mutants vs. wild-type (*), and between +N and -N cells (#), accordingly. Scale bars, 100 nm and 2 μm, in (a) and (c), respectively.

Analysis of ultrastructure images of *N. salina* cells revealed a 37.3% reduction in cell wall thickness in the *cesA1-111* KO mutant, and unchanged cell wall thickness in *cesA2-49* KD and *cesA4-12* KO cells (Figure 1a,b and Supplementary Figure S3a,b). Consistently, visualization of cellulose by CW staining and quantification of cellulose in the *cesA1-111* mutant revealed ca. 80% and 60% reduction in staining intensity and content, respectively, because of the reduced cellulose content (Figure 1c,d). The thinned cell wall observed in the *cesA1* mutant remained thinned even under N-deprivation conditions, while WT and *cesA2* and *cesA4* lines had 1.2- to approximately1.9-fold thicker cell wall with increased cellulose content under these conditions (Figure 1). Hence, CesA1 appears to play a major role in cellulose metabolism in *N. salina*.

### 3.3. Growth and Photosynthesis of cesA1-111 Mutant Are Comparable to Those of the WT

Photosynthetic activities of the *cesA1-111* mutant, as assessed by measuring photosynthetic O_2_ evolution rates at varying light intensities and maximal photochemical efficiency of the photosystem II (Fv/Fm), were comparable to those of WT cells. This ruled out the possibility that *CesA* impairment induces feedback inhibition of the photosynthetic apparatus (Table 1). Consistently, the ultrastructure of *cesA1-111* and WT cells, including chloroplast thylakoid organization, was similar (Supplementary Figure S4). Accordingly, the specific cell growth rates and photosynthetic pigment contents were also similar in these cells (Table 1). This similarity remained unchanged when both cell lines were exposed to N-depletion stress.

### 3.4. cesA1-111 Mutant Has Reduced Chrysolaminarin and Lipid Contents, but Enhanced Protein Content

**Figure 2:**
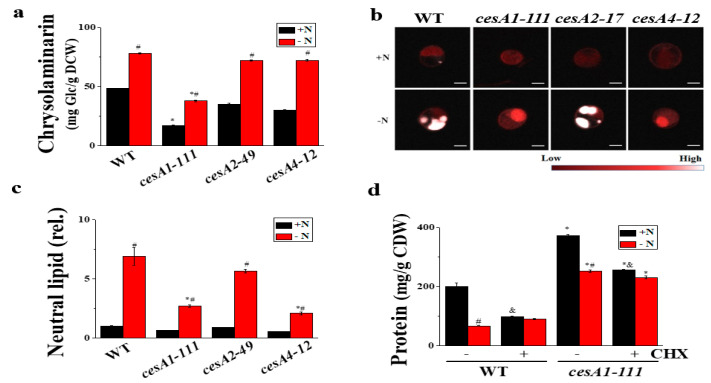
Quantification of chrysolaminarin (**a**), neutral lipid staining with Nile red (**b**) and quantification (**c**), and soluble protein (**d**) content in *N. salina* wild-type (WT) and *cesA* mutants grown under +N and −N conditions for 3 d. In (**b**), the oil bodies in cells stained with Nile red are visible (white) inside the cells. In (**c**), neutral lipid content was determined based on the intensity of Nile red fluorescence at 580 nm (F580) relative to that of N+ WT cells. In (**d**), the translation inhibitor cycloheximide (CHX, 250 μg mL^−1^) was added 2 d prior to the +N or −N growth. Each data point represents the mean ± SE of three biological replicates performed in triplicate. Scale bars, 2 nm in (**b**). The symbols represent statistically significant differences (*p* ≤ 0.05) between mutants vs. WT (*) and between +N and −N cells (#) with CHX (^&^), accordingly.

Considering comparable photosynthetic performance, the hexose P pools in the *cesA1-111* mutant would be utilized for the biosynthesis of chrysolaminarin, lipids, or protein. In fact, in the *cesA1-111* mutant, hexose-P appeared to be used for the synthesis of protein rather than that of the storage C polymers chrysolaminarin and lipid. Both total (soluble and insoluble) chrysolaminarin contents were reduced by 66.3% upon *cesA1* mutation (Figure 2a). The lipid contents were also reduced, as revealed by electron microscopy images of *cesA1-111* cells, with electron-opaque oil bodies occupying a smaller fraction of the cell volume than that in WT cells (Supplementary Figure S4), and by Nile red dye staining (Figure 2b). The dye is used to determine neutral lipids [26] in a concentration-proportional manner [19]. The yellow fluorescence emission of stained mutant cells was ca. 2.7-fold lower (Figure 2c). Instead of chrysolaminarin and oil bodies, the protein content was clearly increased upon *cesA1* mutation (*ca.* 1.9-fold) (Figure 2d). The increase in protein content was either associated with enhanced protein biosynthesis or reduced protein degradation, as cycloheximide treatment significantly inhibited the increase in protein content, while at the same time resulting in increased total protein levels (Figure 2d). Hence, in *N. salina*, impairment of the cellulose biosynthesis appears to facilitate C flux into the protein accumulation.

### 3.5. Hexose-P, Amino Acids, and ROS Contents

**Figure 3:**
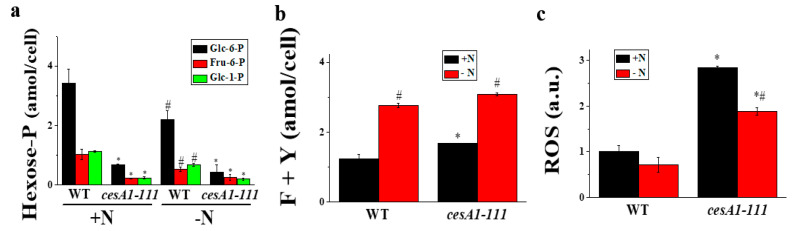
Quantification of hexose-P (**a**), Phe (F) and Tyr (Y) (**b**), and reactive oxygen species (ROS, **c**) contents in *N. salina* wild-type (WT) and *cesA1* mutant grown under +N and −N conditions for 3 d. In (**c**), ROS levels were determined by fluorescence intensity of DCFH-DA at 520 nm (F520) relative to that in +N WT cells. Each data point represents the mean ± SE of three biological replicates performed in triplicate. The symbols represent statistically significant differences (*p* ≤ 0.05) between mutants vs. WT (*), and between +N and −N cells (#), accordingly.

Inhibition of cellulose biosynthesis at the CesA1 catabolizing step would affect the content of its immediate substrate UDP-Glc and the upstream intermediates, hexose-Ps, such as Glc-6-P, Fru-6-P, and Glc-1-P. As expected, the level of these upstream metabolites were significantly reduced upon *CesA1* mutation, and 80–90% lower than those in WT cells (Figure 3a). Further, the amino acid contents were significantly reduced by the *CesA1* mutation, with the exception of Tyr and Phe, whose levels remained almost unchanged (Supplementary Table S3 and Figure 3b). The *CesA1* mutation also affected ROS levels, which were increased ca. 3-fold in the *cesA1-111* KO line (Figure 3c). The *CesA1* mutation induced the reduction of hexose-P pools, or enhancement of Phe and Tyr levels. The ROS levels were not affected by N-deprivation.

### 3.6. Transcript Levels of Genes Involved in Chrysolaminarin, Lipid, and Protein Biosynthesis

**Figure 4:**
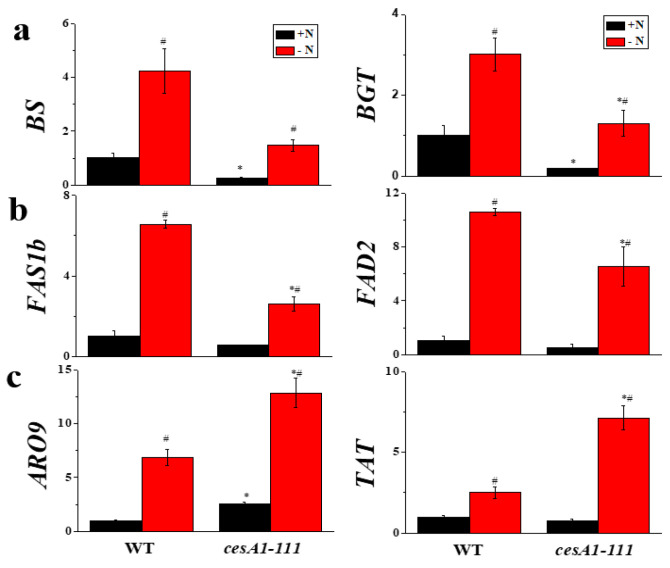
Transcript levels of genes for the biosynthesis of chrysolaminarin (**a**), cytosolic fatty acids and lipids (**b**), and amino acids (**c**) in *N. salina* wild-type (WT) and *cesA1* mutant grown under +N and −N conditions for 3 d. *BS*, 1,3-*β*-glucan synthase; *BGT*, *β*-1,3-glucosyltransferase; *FAS1b*, type I fatty acid synthase; *FAD2*, Δ12 desaturase; *TAT*, tyrosine aminotransferase; *ARO9*, aromatic amino acid aminotransferase. Gene expression (2^−∆∆Ct^) was normalized to that of the housekeeping gene, ubiquitin. Each data point represents the mean ± SE of three biological replicates performed in triplicate. The symbols represent statistically significant differences (*p* ≤ 0.05) between mutants vs. WT (*), and between +N and −N cells (#), accordingly.

Genes for carbohydrates and neutral lipid biosynthesis are transcriptionally activated in response to N-deprivation in *N. salina* [9]. In the current study, impairment of cellulose biosynthesis resulted in a significant reduction in transcript levels for these genes, to different extents (Figure 4). Chrysolaminarin biosynthesis consists of two steps, the first catalyzed by 1,3-*β*-glucan synthase (BS) and the second by *β*-1,3-glucosyltransferase (BGT). The transcript levels of their genes were significantly lowered by *CesA1* mutation under both N-replete and N-depleted conditions (Figure 4a). Among the lipid biosynthesis genes, transcript levels for *FAS1* (*FAS1a* and *FAS1b*) genes encoding cytosolic FAS, a multienzyme complex for the synthesis of palmitic acid (C16:0) and stearic acid (C18:0), and *FAD2* genes encoding the ∆12 desaturase fatty acid desaturase 2 (FAD2) localized in the endoplasmic reticulum [27], were reduced 2-fold by *CesA1* mutation (Figure 4b). However, transcript levels of genes involved in the amino acid Phe and Tyr biosynthesis, including Asp aminotransferase (ARO9) and Tyr aminotransferase (TAT), were almost unaffected by *CesA1* mutation (Figure 4c). However, transcript levels of these genes were increased under N-deprivation (Figure 4a–c).

### 3.7. The Cell Wall of cesA1-111 Mutant Is Prone to Mechanical Stress

**Figure 5:**
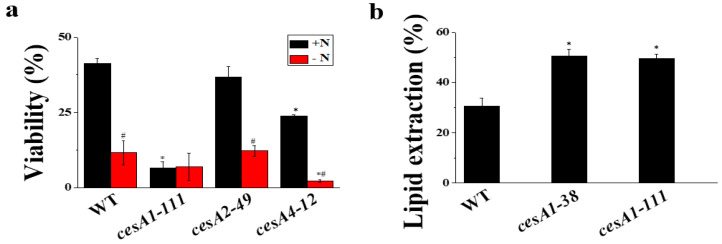
Cell viability after sonication mechanical stress (**a**) and lipid extraction efficiency (**b**) for wild-type (WT) and *cesA* mutants grown under +N (**a**,**b**) and −N (**a**) conditions for 3 d. In (**b**), the lipid extraction efficiency was calculated by dividing the amount of lipids extracted by sonication by total lipid content. Each data point represents the mean ± SE of three biological replicates performed in triplicate. The symbols represent statistically significant differences (*p* ≤ 0.05) between mutants vs. WT (*), and between +N and −N cells (#), accordingly.

Cell wall thickness affects the cell wall breaking force. *N. salina* cells with cell walls thickened in response to N-depletion are more susceptible to mechanical stresses, such as bead beating and sonication, than unstressed cells [9]. In the current study, *cesA1-111* KO mutant with the thinnest cell wall was also most vulnerable to a sonication mechanical stress. The viability of *cesA1-111* KO was reduced by 90% compared with that of WT, while *cesA2-49* and *cesA4-12*, whose wall width was similar to that of the WT, exhibited similar or slightly reduced viability compared with WT (Figure 5a). The lowered resistance to mechanical stress implied that *CesA1* mutation would increase the extracted lipid yield. As anticipated, lipid extraction efficiency from *cesA1-111* mutant was 65% higher than that of WT cells (33% extraction efficiency) (Figure 5b). The lipid extraction efficiency was also higher for the other *cesA1* mutant, *cesA1-38* strain (Figure 5b), strengthening the notion that CesA1-dependent cellulose biosynthesis is indeed important for determining the physico-chemical properties of the cell wall of *N. salina*.

## 4. Discussion

**Figure 6:**
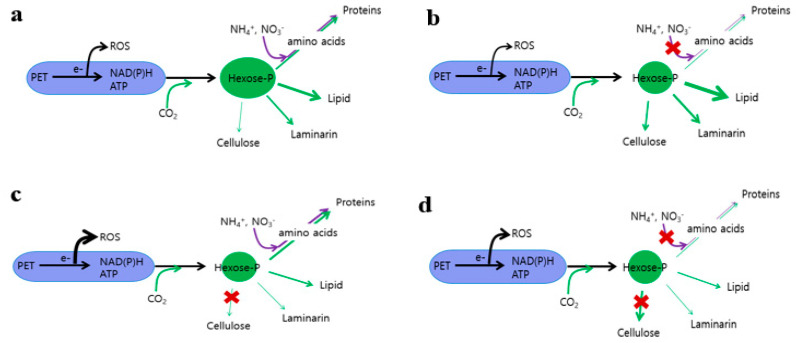
Seesaw model for hexose-P partitioning between C and N polymers in *N. salina*. Partitioning of photosynthetically fixed C into the C and N pathways under normal growth conditions (**a**) is subject to a reciprocal change when one of the pathways is impaired by either nutrient deprivation (**b**) or mutation (**c**). Under N-deficiency conditions, increased conversion of photosynthate assimilates into both storage and structural C polymers, driven by changes in specific gene expression on the transcriptional level, causing reciprocal changes in the protein and C polymer contents (**b**). Genetic impairment of hexose-P partitioning into cellulose biosynthesis appears to favor accumulation of soluble protein rather than storage carbohydrates (**c**). In addition to photochemical reaction, *N. salina* adopts a nonradiative dissipation pathway via reactive oxygen species (ROS), which becomes evident when the availability of the C pathway is reduced (**c**). Simultaneous impairment of the two C and N pathways would involve alternative, but unknown, ROS-independent outlets (**d**).

Photosynthetic light reactions drive the oxidation of water by PSII and photosynthetic electron flow that leads to the generation of chemical energy (ATP), reducing power (NADPH), and reduced electron carriers such as ferredoxins and thioredoxins [1]. In photosynthetic organisms, once produced, these compounds are utilized in anabolic assimilatory pathways for C, N, and S. Imbalance between the production of these energy-rich compounds and their subsequent utilization by anabolic pathways would lead to generation of ROS-dependent damage to photosynthetic apparatus (Figure 6a). In photosynthetic organisms, this adverse situation is minimized by H_2_O -> H_2_O cycle and xanthophyll cycle-dependent nonradiative dissipation [28,29]. Neutral lipid rather than cytosolic chrysolaminarin is a major storage for C polymers in *N. salina* grown under high CO_2_ and N-replete growth conditions [9]. Upon N-deficiency, limitation of amino acids and hence protein biosynthesis (85% reduction) enhances the overall C metabolisms by 1.5–3.0-fold (Figure 6b). Interestingly, these reciprocal changes in C and N polymer ratios are not accompanied by significant changes in ROS levels, indicating that *N. salina* is specialized in handling the imbalance between the photosynthetic light reaction and subsequent C and N metabolism toward lipid production over nonassimilatory outlets. By contrast, imbalance induced by a genetic impairment of the cellulose biosynthesis pathway (*CesA1* mutation) leads to not only protein accumulation instead of storage C polymers accumulation, but also to ROS generation (Figure 6c). By contrast, intracellular ROS accumulation was induced when imbalance between the production of energy-rich compounds and their utilization by C and N metabolisms was simultaneously created via N-deprivation and *CesA1* mutation (Figure 6d).

Generally, excessive production of photosynthetic reductants and chemical energy over their consequent utilization leads to transient ROS generation. These ROS either damage the photosynthetic apparatus [1] or act as signaling molecules for lipid accumulation in oleaginous algae and yeast [29]. In the present study, ROS levels in *N. salina* were not affected by N-deficiency (Figure 3c) with lipid accumulation but were enhanced upon inhibition of cellulose biosynthesis with protein accumulation (Figure 2). Hence, unlike in *Saccharomyces cerevisiae*, where ROS signaling appears to be involved in endoplasmic reticulum stress dependent lipid droplet formation [30], increased ROS in the *N. salina cesA1* mutant would trigger soluble protein accumulation (Figure 2d). This protein accumulation could be either associated with the stimulation of protein biosynthesis or with the inhibition of protein degradation. Partial inhibition of the increase in soluble protein levels induced by CesA deficiency by cycloheximide treatment implies that ROS might control protein stability, which needs further investigation. ROS accumulated in the *cesA1* mutant appear to act as a potent route to eliminate excess electrons. Consistently, a significant increase in ROS levels did not cause appreciable cell damage, since growth rate and photosynthetic performance of the mutant were comparable to those of WT cells (Table 1). The comparable total sum of C and N macromolecules (Figure 1 and Figure 2) and nonradiative dissipation of the absorbed light in WT and *cesA1* mutant cells (Table 1) also support the notion that seesawing of the fixed C allocation between C and N products does not waste fixed carbons via nonradiative outlets nor impair the photosynthetic apparatus.

Lipid metabolism in the oleaginous, *γ*-linolenic acid-producing filamentous fungus *Mucor circinelloides* is enhanced by Tyr-dependent activation of the lipid biosynthesis pathway [31]. Similarly, lipid accumulation is regulated at the transcriptional level in *N. salina* under N-limited conditions [9]. Hence, signaling molecule(s) that regulate fixed C allocation between storage carbohydrates and lipids in *N. salina* are likely amino acids Phe and its hydroxylated form, Tyr, whose levels are increased under both N and C metabolism-limited conditions. However, that signaling is somewhat different from the green alga *C. reinhardtii* and diatom *Phaeodactylum tricornutum* as these algae have different regulatory mechanisms for fixed C allocation between C and N anabolisms. For instance, *C. reinhardtii* with C allocation to structural and storage carbohydrates [28] uses the target of rapamycin to control the C-to-N balance in the cell, regulating growth and biomass accumulation [32]. On the other hand, *P. tricornutum* [33] and *Thalassiosira pseudonana* [34] rely on Gln synthetase/Glu synthase and fructose 2,6-bisphosphatase as C partitioning regulators. Further molecular genetic studies should test whether enzymes related to Phe and Tyr metabolism, including aromatic amino acid aminotransferase, arogenate dehydrogenase, and histidinol-phosphate aminotransferase, act as photoassimilate partitioning regulators or not.

Cell disruption is an expensive step during lipid extraction for algal biodiesel production [13,15]. Further, genetic manipulation of cellulose biosynthesis, which determines cell wall thickness, is a major barrier to cell bioengineering for facile lipid extraction from algae [6]. In the present study, cell wall thinning was achieved by *CesA1* mutation, which also rendered *N. salina* more susceptible to mechanical stress, similar to that observed in cells with thicker cell walls induced by N-deficiency [9], and with a more efficient lipid extraction yield. Hence, CRISPR/Cas-based *CesA* mutation combined with varying culture conditions, such as light intensity, temperature, salinity, and nutrients, is a useful potential target for developing microalgal-based biofuel production.

In conclusion, we here demonstrated a successful CRISPR/Cas genetic manipulation of *N. salina* impacting the cell wall thickness. The cells with thinner cell walls were less resistant to the mechanical stress, which allowed an increased lipid extraction yield compared to the WT cells. The C and N partitioning seesaw model, in combination with the nonassimilatory dissipation proposed for *N. salina* (Figure 6), can guide genetic engineering for *N. salina*-based ideal microalga with enhanced TAG productivity and cost-effective cell wall breakage for bioenergy generation, or the production of some useful proteins to satisfy the demands for pharmaceuticals and other resources.

## Figures and Tables

**Table 1:** Changes in the specific growth rate (SGR, d^−1^), cell size, chlorophyll *a* (Chl) and total carotenoid (Car) content (fg cell^−1^), effective quantum yield of photosystem II (ФPSII), nonradiative dissipation of the absorbed light via heat (NPQ), and O_2_ evolution rate (mmolO_2_ g Chl^–1^ h^–1^) under white-light illumination (500 μmol m^−^^2^s^−^^1^) in *N. salina* wild-type (WT) and *cesA1* mutant grown under +N and –N conditions for 2 d. Data are expressed as the average of three biological replicates ± SE. The symbols represent statistically significant differences (*p* ≤ 0.05) between mutant vs. WT cells (*), and between +N and -N cells (#), accordingly.

Strain	SGR	Chl	Car	ФPSII	NPQ	O_2_ Evolution
WT						
+N	0.14 ± 0.01	203.18 ± 2.14	30.23 ± 1.61	0.58 ± 0.03	1.58 ± 0.32	0.34 ± 0.04
−N	0.05 ± 0.02 ^#^	112.67 ± 3.28 ^#^	19.38 ± 0.79 ^#^	0.49 ± 0.01 ^#^	3.09 ± 0.02 ^#^	0.41 ± 0.02
*cesA1-111*						
*+*N	0.11 ± 0.01 *	172.36 ± 8.46 *	28.86 ± 2.70	0.59 ± 0.02	1.70 ± 0.30	0.37 ± 0.02
−N	0.03 ± 0.01 ^#^	90.16 ± 2.5 *^,#^	17.15 ± 0.35 ^#^	0.52 ± 0.02	3.38 ± 0.0 ^#^	0.41 ± 0.01

## Supplementary Materials

The following Supplementary Materials are available online at https://www.mdpi.com/2076-2607/8/8/1195/s1. Figure S1: Schematic maps of pNsCas9 and sgRNA expression, and pNsCesAs-RNAi vectors targeted to CesA1-4 genes. Figure S2: Generation of cesA knockout and knockdown mutants. Figure S3: Calcofluor white (CW) (a,b) and Nile red (c,d) fluorescence images (a,c) and quantification (b,d) of stained N. salina wild-type (WT) and Crispr/Cas9 knockout(cesA1-38) mutant cells grown under +N and -N conditions for 3 d. Figure S4: TEM images of N. salina wild-type (WT) and cesA mutant cells grown under +N and -N conditions for 3 d. Table S1: Primer sequences for cloning and Northern blot. Table S2: Primers used for qRT-PCR analysis. Table S3: Amino acid content of N. salina wild-type (WT) and cesA1 mutant cells grown under +N and -N conditions for 2 d.
