# Supplementary material for: Genetic Impairment of Cellulose Biosynthesis Increases Cell Wall Fragility and Improves Lipid Extractability from Oleaginous Alga Nannochloropsis salina
Source: Microorganisms. 2020 Aug 6;8(8):1195. doi: 10.3390/microorganisms8081195 (PMC7464416; doi:10.3390/microorganisms8081195)
Supplement: Supplementary file 1 [file microorganisms-08-01195-s001.pdf]

Supplementary Materials

Genetic Impairment of Cellulose Biosynthesis Increases Cell Wall Fragility with Enhanced Lipid Extractability in Oleaginous Alga *Nannochloropsis salina*

Seek Won Jeong[1,†], Kwon HwangBo[2,†], Jong Min Lim[2], Seung Won Nam[3], Bong Soo Lee[4], Byeong-ryool Jeong[5], Yong Keun Chang[,6], Won-Joong Jeong[2,*], Youn-Il Park[1,*]

1 Department of Biological Sciences, Chungnam National University, Daejeon 34134; neditbe@cnu.ac.kr
2 Korea Research Institute of Bioscience and Biotechnology, Daejeon 34141, Korea; rnjsl@kribb.re.kr (K.H.); jmlim114@kribb.re.kr (J.M.L.)
3 Bioresouces Culture Collection Division, Nakdonggang National Institute of Biological Resources, Sangju 37242, Korea; seungwon10@nnibr.re.kr
4 Department of Microbial and Nano Materials, College of Science and Technology, Mokwon University, Daejeon 35349, Korea; bongsoolee@mokwon.ac.kr
5 Single-Cell Center, CAS Key Laboratory of Biofuels and Shandong Key Laboratory of Energy Genetics, Qingdao Institute of BioEnergy and Bioprocess Technology (QIBEBT), Qingdao 266101, China; bjeong@unist.ac.kr
6 School of Energy and Chemical Engineering, Ulsan National Institute of Science and Technology (UNIST), Ulsan 44919, Korea
7 Department of Chemical and Biomolecular Engineering, Korea Advanced Institute of Science and Technology, Daejeon 34141, Korea; changyk@kaist.ac.kr
* Correspondence: wonjoong@kribb.re.kr (W.J.J.); yipark@cnu.ac.kr (Y.-I.P.); Tel.: +82-42-860-4468 (W.J.J.); +82-42-821-5493 (Y.-I.P.)
† These authors contributed equally to this work

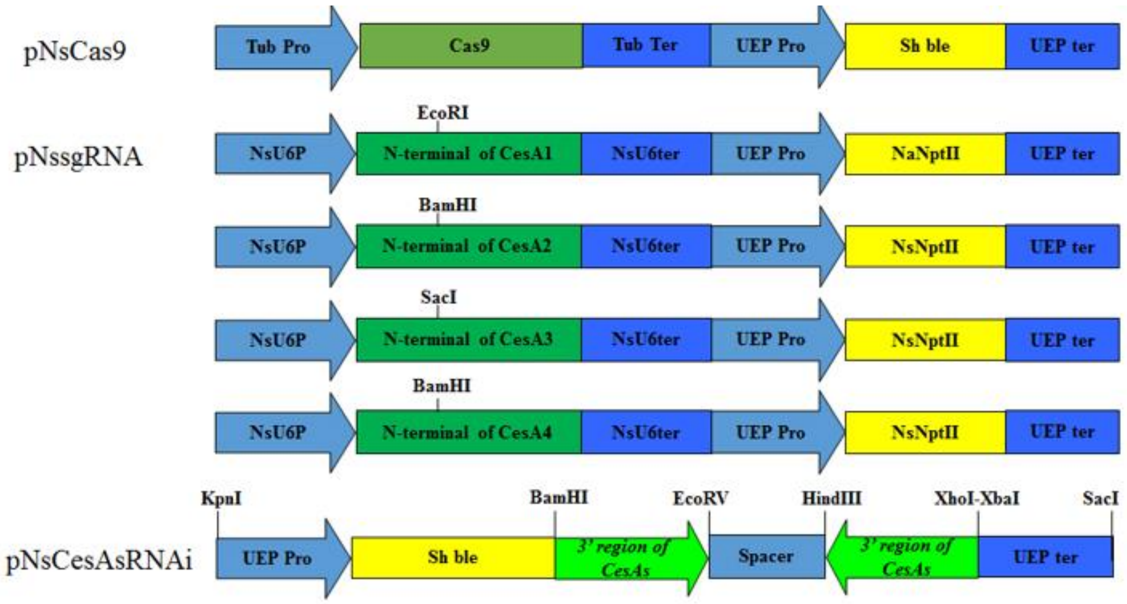

Figure S1. Schematic maps of pNsCas9 and sgRNA expression, and pNsCesAs-RNAi vectors targeted to *CesA1-4* genes. Bars indicate target positions with the EcoRI, BamHI, and SacI restriction sites, accordingly.

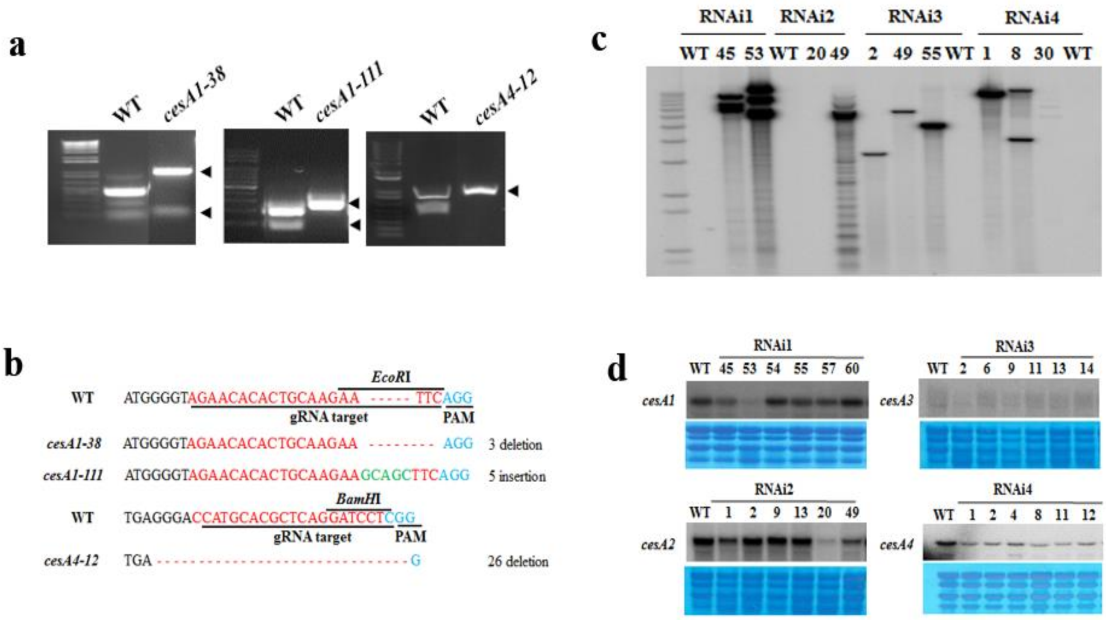

Figure S2. Generation of *cesA* knockout and knockdown mutants.

(a) Confirmation of knokout lines by *in vitro* cleavage of PCR-amplified *CesA* using EcoRI (*cesA1-38* and *-111*) and BamHI (*cesA4-12*) restriction enzymes. Arrowheads indicate PCR-amplified fragments (498 and 482 bp, 200 and 100 bp, and 395 and 426 bp) specific for the respective cell lines (*cesA1-38*, *cesA1-111*, and *cesA4-12*, accordingly).

(b) CRISPR/Cas9 generated small indels at the target sites (the *CesA1* and *CesA4* loci, accordingly).

(c) Southern blot analysis confirming transgene integration into one or several loci in the genome in the *cesA1–4* (RNAi1–4) knockdown cells.

(d) Northern blot analysis showing variable expression of the *CesA1*, *CesA2*, and *CesA4* genes in *cesA1*, *cesA2*, and *cesA4* knockdown lines, accordingly. Equal RNA loading was checked by methylene blue staining (bottom).

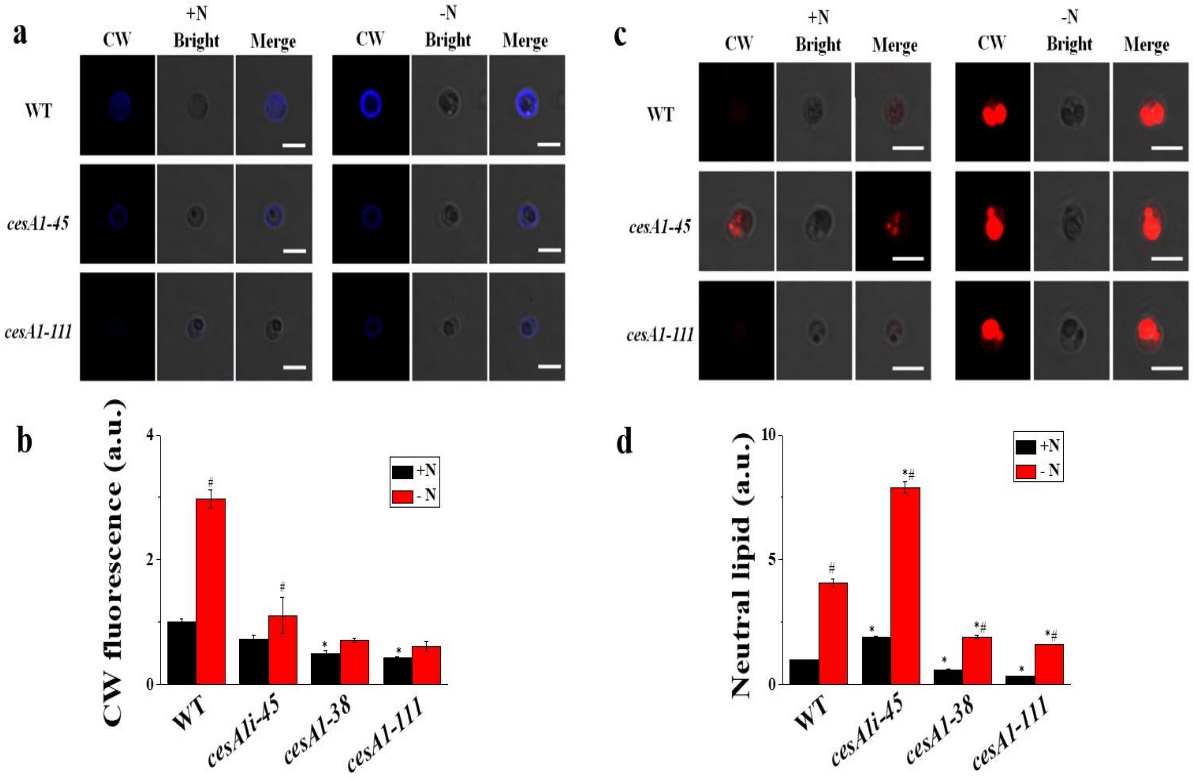

Figure S3. Calcofluor white (CW) (a, b) and Nile red (c, d) fluorescence images (a, c) and quantification (b, d) of stained *N. salina* wild-type (WT) and Crispr/Cas9 knockout *(cesA1-38)* mutant cells grown under +N and -N conditions for 3 d. The *cesA1-111* mutant was used for comparison. Neutral lipid and cellulose contents were estimated by Nile Red fluorescence amplitude at 580 nm (F_{580}) and calcofluor white amplitude at 420 nm (F_{420}) relative to those of WT before N– induction, respectively. Each data point represents the mean ± SE of three biological replicates performed in technical triplicate. Symbols represent statistically significant differences ($P \leq 0.05$) between mutants *vs* wild type (*), and between +N and -N cells (#), respectively.

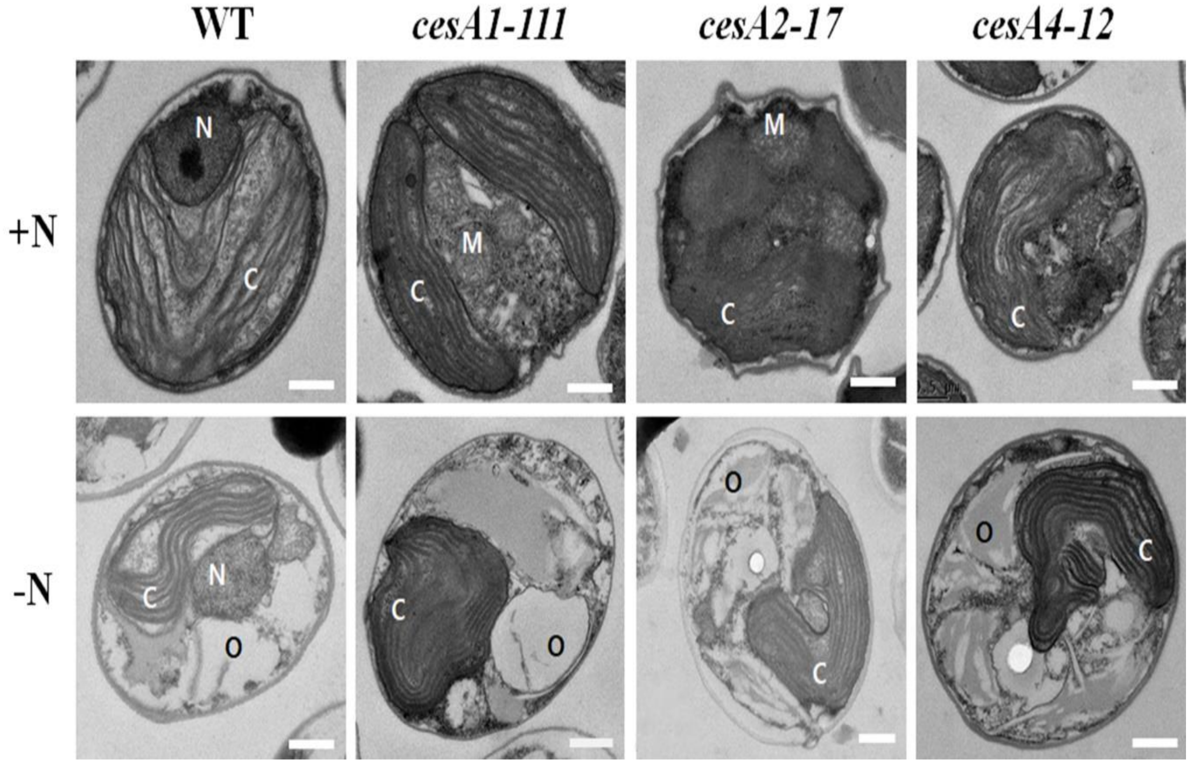

Figure S4. TEM images of *N. salina* wild-type (WT) and *cesA* mutant cells grown under +N and -N conditions for 3 d.

Table S1. Primer sequences for cloning and Northern blot.

Gene	Primer	Sequence (5′ to 3′)	Purpose
Spacer	Spacer-F	AAAGATATCGTGATAATTCAGATGTAGGA	Cloning
	Spacer-R	TTTAAGCTTAGATAATTCGTGATGCATCCT	
CesA1	CESA1-F	GGATCCACTCGAGTCTCTCCAGGAGTCCGTCT	cDNA cloning for RNAi
	CESA1-R	GATATCAAGCTTAGCGGAGGAGATAGCCAATA	
	CESA1-F(gib)	GTAGCATGGTATCCATCTAATTGAAATTTACATCAAGCTC	Gibson assembly
	CESA1-R(gib)	GAGCTTGATGTAAATTTCAATTAGATGGATACCATGCTAC	
	CESA1-2-F1	CCTTCATCATCGCCGAGACCATGA	gDNA PCR
	CESA1-2-R1	TGTGCCGGACGAAGATCTCCA	
	CESA1-2-F2	TGAACGAGGGCACAAGTGGTGA	Probe for northern blotting
	CESA1-2-R2	GTCCCAGCCGTGGAGCACA	
CesA2	CESA2-F	GGATCCACTCGAGCGGGATGTGCCCAAGCTGT	cDNA cloning for RNAi
	CESA2-R	GATATCAAGCTTAGGGTACAGCTGCCAGAGCA	
	CESA2-F(gib)	GTAGCATGGTATCCATCTAAAGACCATAATGCAGCCTTAC	Gibson assembly
	CESA2-R(gib)	GTAAGGCTGCATTATGGTCTTTAGATGGATACCATGCTAC	
	CESA2-2-F1	CATTCGGAGACCATTCCAAACGG	gDNA PCR
	CESA2-2-R1	TCATCGCAAATGTTGACCGTGATCT	
	CESA2-3-F1	CGGGATGTGCCCAAGCTGTATT	Probe for northern blotting
	CESA2-3-R1	GTTTCTCCCGCGTGAATGTCGGGA	
CesA3	CESA3-F	AAGGATCCACTCGAGCTTCCCTTCCATCTGGCA	cDNA cloning for RNAi
	CESA3-R	TTGATATCAAGCTTAGGAGGAGACGAAGGCGTA	
	CESA3-2-F1	CTTCATCTCCTCTGGCTCCAAGCT	gDNA PCR
	CESA3-2-R1	GACGGTCATCCACAACCACCAA	
	CESA3-3-F1	GCTTCCCTTCCATCTGGCA	Probe for northern blotting
	CESA3-3-R1	ATGCGTTGGAAGCAGGAGGA	
CesA4	CESA4-F	GGATCCACTCGAGTCAGCTTCGCCACGGCCTT	cDNA cloning for RNAi
	CESA4-R	GATATCAAGCTTGCGTATCGGAGGTAGAAGCT	
	CESA4-F(gib)	GTAGCATGGTATCCATCTAATGTCTCATCTGTTTCTTGCA	Gibson assembly
	CESA4-R(gib)	TGCAAGAAACAGATGAGACATTAGATGGATACCATGCTAC	
	CESA4-2-F1	GGGACCCCTTACCTTCCCTTGA	gDNA PCR
	CESA4-2-R1	CACCAAATGTCGTGCATGTCCGA	
	CESA4-3-F1	TTCCGATCCGTCCAGCTCAGCTT	Probe for northern blotting
	CESA4-3-R1	GCCGCGTATCGGAGGTAGAAGCT	
NsU6 promoter	NsU6P-F	AACCCGGGGTCTGAGTACGTCTCCGAAA	gDNA cloning
	NsU6P-R	CGAGTTAGATGGATACCATGCTACT	

	NsU6P-F(gib)	TATAGGGCGAATTGGGTACCCCCGGGGTCTGAGTACGTCT	Gibson assembly
	NsU6P-R(gib)	AGACGTACTCAGACCCCGGGGGTACCCAATTCGCCCTATA	
NsU6 terminator	NsU6ter-F	AAAGATATCGTGATAATTCAGATGTAGGA	gDNA cloning
	NsU6ter-R	TTTAAGCTTAGATAATTCGTGATGCATCCT	
ble	Ble-F1	GCCAAGTTGACCAGTGCCGTT	Probe for Southern blotting
	Ble-R1	TGCTCGCCGATCTCGGTCAT	

Table S2. Primers used for qRT-PCR analysis.

Gene	Description	Forward primer (5′ to 3′)	Reverse primer (5′ to 3′)	Reference (GenBank)
Bs	1,3-β- Glucan synthase	GGCGTGGTAATAAGCGAC	GTTCAGTAAAGCTGATGCC	AFGQ01002722
Bgt	β-1,3-Glucosyltransferase	ATGTCTTGGAGGCCATGGAG	TTTGCTCGTGGTCGTCAGC	AFGQ01002665
Fas1b	Type I fatty acid synthase	TTGCAGCGTGTAGTGCTG	AAGCATCTCCCAAAAAGCC	KK037387
Fad2	Δ12 desaturase	CACCCTTGCTGACATCAAAG	CGCATTCATGGGCAATCAC	AFGQ01000627
AlaDH	Alanine dehydrogenase	AACAGCATGCTCACTACC	CCGTGGGATAAGAGGATC	
Asa	Argininosuccinate lyase	GACTTGCAACGTCTGGTG	AACGACACGCCTGCAAAG	
Asns	Asparagine synthetase	GCGGTATGTTCTCTTTCG	AGCGCCTTCATTTCACTC	
Aspat	Aspartate aminotransferase	AAGGTGTTAGAGGCGAAG	GCCACACGCTTTTCTTTC	
Gs	Glutamine synthetase	CTGCCACACGAATTTCTC	GTCAGTCGACGCTCGTTC	
Ghmt	Glycine hydroxymethyltransferase	TGCGATGTCCTGAACATC	AGCCACCTGCTCGAAATC	
Hisd	Histidinol dehydrogenase	TTGAAGGGTTTTGCCGTC	AAAACGGCTCCGTAGTTC	
Ms	Methionine synthase	CTAGTAAGTTGTGTGCGG	ATGGTCAGTAAGCCTTGC	
Ast2	Aspartate aminotransferase	TGCGAGTACTGCGATAAG	ATGACCACAACATTGTCTG	
Pycr	Pyrroline-5-carboxylate reductase	CATCGTGTTCGAATCTCG	CGGTGGAGCACTTGATTG	
Psph	Phosphoserine phosphatase	TCCAAGACGCGTTGAAAG	AGACCAAATAGACCGCTG	
Thrs	Threonine synthase	GATATGTTGGATGCGCAC	TCCAGCTCTCTCAATTGG	
TrpA	Tryptophan synthase	GCTTTCTACCGGAAACAG	GTTGATTTTATGCGCGCC	
Tat	Tyrosine aminotransferase	GGCTGGAAAATCTCACTC	TCGACCAAGATACTGACG	
Aro9	Aromatic amino acid aminotransferase	ATCCACCTAGAGTCTCTC	GACCGTGCAGGGAAATTG	
Ubq	Ubilquitin	GGCAAGACGATCACACTGGA	AAAGCGCGTCTCCACCAC	AFGQ01000799

Table S3. Amino acid content of *N. salina* wild-type (WT) and *cesA1* mutant grown under +N and –N conditions for 2 d. Data are expressed as the average of three biological replicates performed in triplicate (amol $cell^{-1}$) ± SE. The symbols represent statistically significant differences ($P \leq 0.05$) between mutants *vs* WT (*), and between +N and -N cells (#), accordingly.

Amino acid	WT		*cesA1-111*	
	+N	-N	+N	-N
Ala	34.90 ± 7.50	4.94 ± 1.43#	4.92 ± 0.32*	4.84 ± 0.21
Arg	36.62 ± 9.28	1.78 ± 0.15#	4.48 ± 0.28*	1.17 ± 0.16*#
Asn	4.11 ± 1.07	0.89 ± 0.20#	0.30 ± 0.18*	0.56 ± 0.06
Asp	12.66 ± 9.94	1.12 ± 0.06#	1.29 ± 0.05*	0.77 ± 0.06
Gln	20.19 ± 2.56	0.95 ± 0.01#	10.19 ± 1.75*	3.72 ± 0.07*#
Glu	98.65 ± 8.64	9.32 ± 2.08#	6.21 ± 0.43*	2.98 ± 0.71* #
Gly	2.78 ± 0.12	1.37 ± 0.03#	1.13 ± 0.10*	0.90 ± 0.12
His	2.80 ± 1.44	0.48 ± 0.03#	0.14 ± 0.05*	0.20 ± 0.02*
Ile	1.32 ± 0.50	1.16 ± 0.06	0.36 ± 0.09*	0.87 ± 0.14#
Leu	2.74 ± 0.95	2.75 ± 0.29	1.01 ± 0.26*	3.02 ± 0.23*#
Lys	8.89 ± 0.17	5.62 ± 0.76#	1.21 ± 0.41*	3.37 ± 0.20#
Met	0.62 ± 0.06	0.77 ± 0.08	0.05 ± 0.01*	0.43 ± 0.06#
Phe	0.68 ± 0.20	1.75 ± 0.08#	1.01 ± 0.25	2.07 ± 0.08#
Pro	27.68 ± 7.76	2.66 ± 0.39#	1.32 ± 0.66*	1.34 ± 0.24*
Ser	4.07 ± 0.62	1.51 ± 0.20#	0.69 ± 0.18*	1.31 ± 0.04#
Thr	1.57 ± 0.57	1.21 ± 0.20 [a]	0.42 ± 0.05*	0.81 ± 0.10#
Trp	0.46 ± 0.19	0.47 ± 0.08 [a]	0.10 ± 0.04*	0.37 ± 0.05#
Tyr	0.55 ± 0.06	1.01 ± 0.02 [a,*]	0.67 ± 0.24*	1.01 ± 0.12
Val	6.65 ± 2.58	3.42 ± 0.76 [a]	1.20 ± 0.09*	2.54 ± 0.12#
